# A first AFLP-Based Genetic Linkage Map for Brine Shrimp *Artemia franciscana* and Its Application in Mapping the Sex Locus

**DOI:** 10.1371/journal.pone.0057585

**Published:** 2013-03-04

**Authors:** Stephanie De Vos, Peter Bossier, Gilbert Van Stappen, Ilse Vercauteren, Patrick Sorgeloos, Marnik Vuylsteke

**Affiliations:** 1 Laboratory of Aquaculture, Artemia Reference Center (ARC), Department of Animal Production, Ghent University, Gent, Belgium; 2 Department of Plant Systems Biology, VIB, Gent, Belgium; 3 Department of Biotechnology and Bioinformatics, Ghent University, Gent, Belgium; Auburn University, United States of America

## Abstract

We report on the construction of sex-specific linkage maps, the identification of sex-linked markers and the genome size estimation for the brine shrimp *Artemia franciscana*. Overall, from the analysis of 433 AFLP markers segregating in a 112 full-sib family we identified 21 male and 22 female linkage groups (2*n* = 42), covering 1,041 and 1,313 cM respectively. Fifteen putatively homologous linkage groups, including the sex linkage groups, were identified between the female and male linkage map. Eight sex-linked AFLP marker alleles were inherited from the female parent, supporting the hypothesis of a WZ–ZZ sex-determining system. The haploid *Artemia* genome size was estimated to 0.93 Gb by flow cytometry. The produced *Artemia* linkage maps provide the basis for further fine mapping and exploring of the sex-determining region and are a possible marker resource for mapping genomic loci underlying phenotypic differences among *Artemia* species.

## Introduction

*Artemia*, known as brine shrimp, is a genus of small planktonic crustaceans found worldwide in natural salt lakes and salterns [1]. Their larvae (nauplii) are the most commonly used live food in aquaculture activities, specifically for larval growth of more than 85% of the marine species reared in aquaculture [2], [3]. Adult *Artemia* survive extreme salinities, while their encysted embryos (cysts), produced under stressful conditions, have a unique tolerance for high doses of UV and ionizing radiation, anoxia, thermal extremes and desiccation-hydration cycles [4]–[6]. Cysts remain viable for years and produce nauplii within 24 h after hydration.

An overview of *Artemia* cytogenetics, DNA content and available molecular tools is provided. Six different sexually dimorphic species can be found in the *Artemia* genus, among which *Artemia franciscana Kellogg*, 1906 [4] and several obligate parthenogenetic *Artemia* populations ranging in ploidy from 2n to 5n [7]. All sexually dimorphic *Artemia* species are diploids with 2n = 42, except *A. persimilis* (2n = 44) [8]. The *Artemia* genome size has been assessed with two different techniques producing discordant estimates: 2.93 Gb (3 pg) by Feulgen densitometry [9] and 1.47 Gb (1.5 pg) by DNA reassociation kinetics [10]. Despite the use of flow cytometry in the most recent evaluations of crustacean genome size [11]–[13], so far no flow cytometry-based estimates of the *Artemia* genome have been published. To date, genomic resources for *Artemia* have been limited to RAPD [14], [15], RFLP [16], AFLP [17], [18], microsatellite markers [19] and the 15,822 bp mitochondrial genome sequence [20], [21].

In crustaceans, three major genetic sex determination systems have been suggested by cytogenetics and sex-linked markers: WZ-ZZ (females are the heterogametic sex), XX-XY (males are the heterogametic sex) and androdioecy (a mix of ZZ males and WZ hermaphrodites, as in *Eulimnadia texana*) [22]. Examples of crustaceans with an XX-XY sex-determining system are decapods such as the Chinese mitten crab *Eriocheir sinensis* [23], [24], terrestrial isopods and the amphipods *Orchestia cavimana* and *O. gammarellus* [25]. However, a WZ-ZZ sex-determining system has been found in decapods such as *Litopenaeus vannamei* [26], tiger shrimp *Penaeus monodon* [27], *Macrobrachium rosenbergii* [28], kuruma prawn *Penaeus japonicus* [29], Australian red claw crayfish *Cherax quadricarinatus* [24] and in isopods like *Armadillidium vulgare* and all Valvifera, except *Saduria entomon* [30]. In bisexual *Artemia*, female heterogamety has been suggested previously by observation of sexual heterochromosomes in *A. salina* [31], *A. franciscana* and *A. persimilis* [8]; by crossing experiments with *A. franciscana* showing a recessive sex-linked trait called “white eye” [32] and by karyotyping and heterochromatin experiments showing one heterochromatic block in female and two in male *A. persimilis* [8].

Over the last decade, linkage maps have been developed for a number of crustaceans such as *Daphnia pulex* [33], *D. magna* [34], *Tigriopus californicus* [35], *P. monodon* [36]–[38], *L. vannamei* [39]–[41], *Fenneropenaeus chinensis* 42,43 and *P. japonicus* [44]. Sex-linked markers have been found in males of the isopod *Mysis relicta* [45] and of *Triops cancriformis* [46]. In female crustaceans, sex-linked markers have been found in the isopods *Paracerceis sculpta* [47] and *Jaera ischiosetosa* [48], in the crab *Cancer setosus* [49], in penaeid shrimps *L. vannamei* [41] and *P. monodon* [27] and in giant freshwater prawn *M. rosenbergii* [50]. Moreover, a hermaphrodite-determining allele has been studied in the androdioecious branchiopod *E. texana* [51]. So far, neither linkage maps, nor trait-linked markers including sex-linked markers have been identified in *Artemia* [8].

Genetic linkage maps are invaluable in forward genetic analyses for the identification of the genomic loci responsible for phenotypic differences. From this perspective, *Artemia* offers a number of major advantages for time-effective generating of experimental mapping populations and for mapping natural allelic variation. They have a short generation time (2–4 weeks), offspring production of several hundred individuals per brood, storability of cysts for years, easy breeding in large numbers and levels of genetic variability that are among the highest within crustaceans [16], [41], [52]. In addition, we expect that forward genetic approaches in *Artemia* are not only restricted to *Artemia*-specific traits, but are also valuable for mapping traits such as sex, *Vibrio* pathogen resistance and growth rate, segregating in commercially important crustaceans. We believe therefore, that *Artemi*a could be a useful model species for other crustaceans.

In the present study, we report on a first AFLP-based linkage map of *A. franciscana*. We additionally present eight sex-linked markers that disclose the linkage group corresponding to the W chromosome and confirm female heterogamety in *A. franciscana*. Finally, we report on the estimation of the *A. franciscana* genome size by flow cytometry.

## Materials and Methods

### Mapping population

Cyst material of the *A. franciscana* strains from San Francisco Bay, USA (SFB; ARC1364) and Vinh Chau, Vietnam (VC; ARC1349) was obtained from the Laboratory of Aquaculture & Artemia Reference Center cyst bank (http://www.aquaculture.ugent.be). The SFB strain was first introduced in Vinh Chau, Vietnam in 1982, eventually resulting in the new VC strain in the late 1980`s [53]. First, cysts from both strains were hatched separately in aerated seawater (28°C, salinity 35 g.l^−1^). The instar I nauplii of each strain were then harvested and reared for a week in aerated seawater with added sea salt (Instant Ocean®, 28°C, final salinity 70 g.l^−1^) and fed with *Tetraselmis suecica*, a marine unicellular green alga. The *Artemia* were subsequently transferred to individual Falcon tubes and kept there under the same conditions for seven days until sexual maturation. A controlled cross between VC (♀) and SFB (♂) was then made, resulting in F_1_ progeny that was collected over a sieve every two days and grown until maturity under the same conditions as the parental generation. Adult F_1_ progeny was rinsed with sterile distilled water and the phenotypic sex of each F_1_ offspring individual was determined visually. For gut evacuation before DNA extraction, the offspring and parents were starved during 24 h, followed by removal of the brood pouch in females. Parents and progeny were stored individually at −20°C.

### DNA extraction

DNA was extracted from parents and their 112 F_1_ offspring according to a modified CTAB-method for shrimp tissue [54]. Briefly: to each sample, ground in liquid N_2_, 150 µl of CTAB buffer was added. After homogenization, 750 µl of extra CTAB buffer was added and the mix was left at 25°C for 30 min. PCA solution was added (600 µl; 25∶24∶1 phenol/chloroform/isoamylalcohol). After centrifugation, 800 µl of the upper aqueous phase was added to 600 µl of CA solution (24∶1 chloroform/isoamylalcohol) and the mix was homogenized. To 700 µl of the upper aqueous phase, 630 µl of isopropanol was added. The mix was incubated for 1 h at −70°C. After centrifugation, the pellet was washed with 600 µl of ethanol 70%, air-dried in a 60°C oven and resuspended in 20 µl of sterile distilled water. DNA quality and concentration were assessed on a 1% agarose gel.

### Segregation analysis and linkage mapping

AFLP analysis with fluorescent dye detection was performed on a LI-COR long read-IR^2^ 4200 (LI-COR Biosciences) as described by Vuylsteke et al. [55]. Sixty-five *Eco*RI+3/*Mse*I+3 primer combinations (PCs) listed in Table S1 were used. AFLP analysis of parents and 112 offspring was done on two separate 64-lane gels per PC.

The degree of polymorphism between the two parental strains was estimated based on AFLP fragments amplified by four PCs (E112M212, E112M213, E112M233 and E112M234).

AFLP markers were scored using the specific image analysis software AFLP-Quantar*Pro* (http://www.keygene-products.com) as described in Vuylsteke et al. [55]. Each AFLP marker was identified by (1) a code referring to the corresponding PC (Table S1), followed by (2) the molecular size of the fragment in nucleotides as estimated by AFLP-Quantar*Pro*, and (3) a tag referring to the type of marker. Parental AFLP markers segregating 1∶1 in the F_1_ progeny are heterozygous in either the female (female marker, tagged as “F”) or the male parent (male marker, tagged as “M”) and homozygous absent in the other parent. AFLP markers heterozygous in one of the parents and homozygous present in the other were not included in the linkage analysis, because heterozygotes could not be reliably discriminated from individuals homozygous for the “band present” allele. No tag was used for biparental markers, which are heterozygous in both parents and thus, segregate 1∶2∶1 in the F_1_ progeny. Parental and biparental AFLP markers were scored co-dominantly based on relative fragment intensities resulting in more genetic information compared to dominant (present/absent) scoring and hence, speeding up the mapping process [55]. However, biparental markers were scored dominantly when the heterozygotes could not reliably be discriminated from the individuals homozygous for the “band present” allele.

Linkage and segregation analyses were performed using the software package Joinmap 4 [56]. The mapping population type was set to CP (i.e. a population resulting from a cross between two heterogeneously heterozygous and homozygous diploid parents, linkage phases originally unknown). The segregation type was encoded according to Joinmap 4 recommendations [56]. A logarithm of the odds (LOD) threshold range between 2.0 and 14.0 was initially used to group parental markers. Only linkage groups containing at least three markers were considered for map construction. Segregation distortion of markers was tested by using a χ^2^-test as implemented in Joinmap 4. Graphical presentation of linkage groups was done with the software MapChart [57].

### Artemia genome size estimation by flow cytometry

The haploid genome size (GS) of *Artemia* was assessed against the rainbow trout (haploid GS 2.4–3.0 pg or 2.35−2.93 Gb [58]) and the chicken genome (haploid GS 1.07 pg or 1.05 Gb [59]), both used as internal standards.

The consistency of the used method was assessed by calibrating rainbow trout nuclei (2 µl of freshly drawn heparinized *Oncorynchus mykiss* blood) against chicken erythrocyte nuclei (2 µl of 10x diluted BioSure®CEN singlet, *Gallus gallus domesticus*, Rhode Island Red female).

Each of the four *Artemia* individuals (i.e. four full-sib males from the VC (♀) x SFB (♂) cross) were chopped together with internal standard material using a razor in 1 ml of Galbraith`s buffer as described in Dolezel and Bartos [60]. Cell suspensions were filtered through a 30 µm mesh, put on ice and nuclei were co-stained in the dark for 2 min with 50 µl of fluorescent DNA stain Propidium Iodide (Sigma-Aldrich PI solution in water 1 mg/ml). The use of PI staining on *A. franciscana* (GC% 32) [61], *O. mykiss* (GC% 42) [58] and *G. domesticus* (GC% 47) [62] was chosen to avoid a GC content-linked bias, as occurs with DAPI staining [60]. At least 5,000 nuclei were analyzed for each co-stained sample, using a Modular Flow cytometer and cell sorter (MoFlo Legacy, Cytomation) with a 488 nm Argon laser and PI emission bandpass filter of 580/30 nm. Instrument calibration was performed using Flow-check Fluorospheres (Beckman Coulter) and internal standards. Fluorescence of the nuclei was recorded linearly with the software Summit v4.3. For each co-stained sample, fluorescence histograms were generated and mean fluorescence values were calculated with the flow cytometry data analysis software Cyflogic 1.2.1. The haploid GS of for each *Artemia* sample was calculated according to the following formula [13]: GS  = 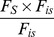, where *F_s_* is the mean fluorescence of the sample and *F_is_* is the mean fluorescence of the internal standard.

## Results

### Segregation analysis and linkage mapping

A total of 65 AFLP PCs resulted in a total of 531 markers, of which 433 were parental (239 female, 194 male) and 98 markers were biparental. Based on only four primer combinations (PCs) yielding 180 AFLP fragments, 36% of the fragments segregated between both parents.

**Figure 1:**
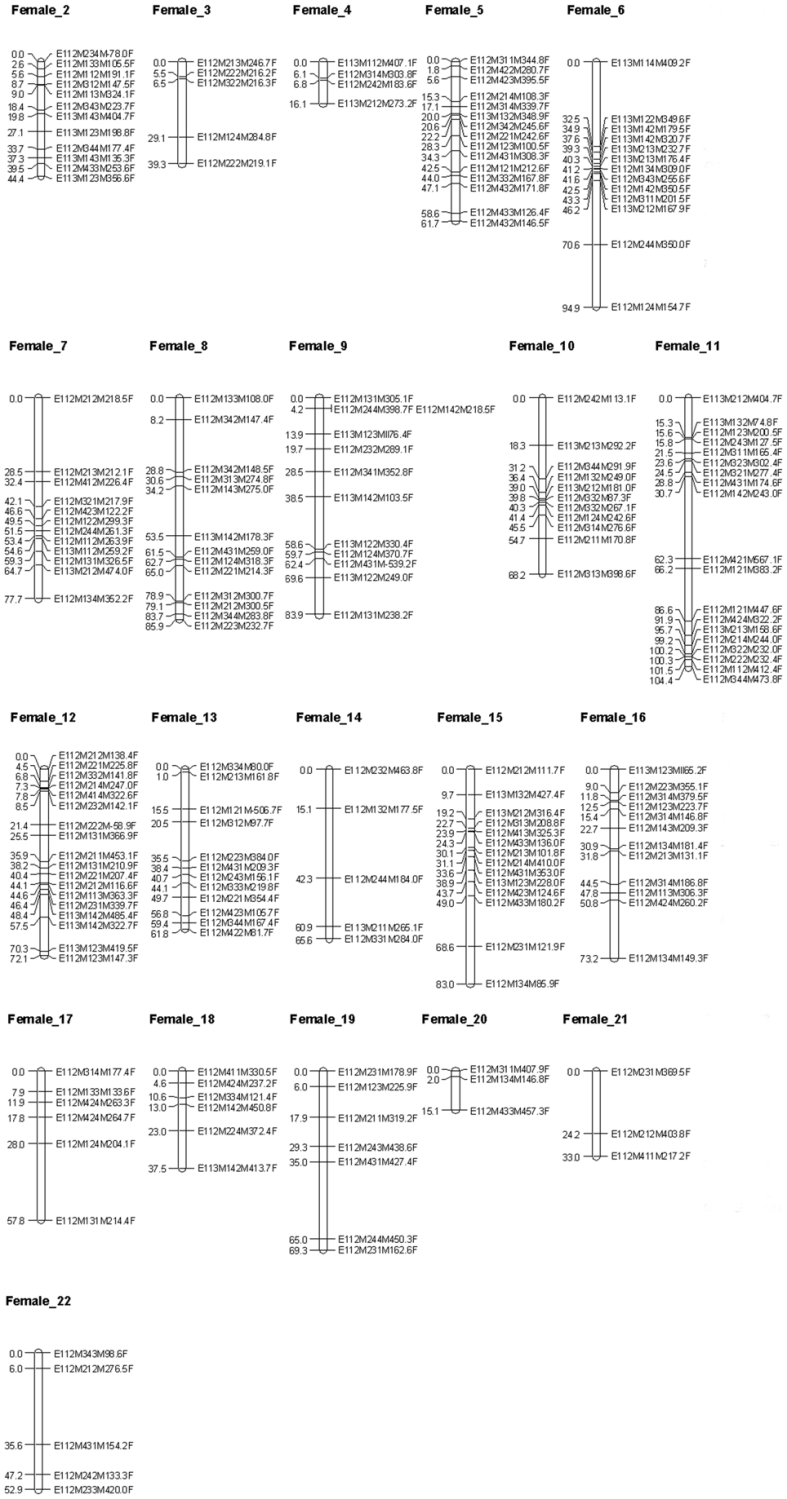
*Artemia franciscana* autosomal female linkage groups Twenty-one linkage groups representing the *Artemia franciscana* autosomal genome containing markers originating from female parental strain Vinh Chau (ARC1349). Each AFLP marker is represented by (1) a code referring to the corresponding PC (Table S1), followed by (2) the molecular size of the fragment in nucleotides and (3) the type of parental marker (female marker, tagged as “F”). Cumulative marker distances (cM) are indicated on the left.

**Figure 2:**
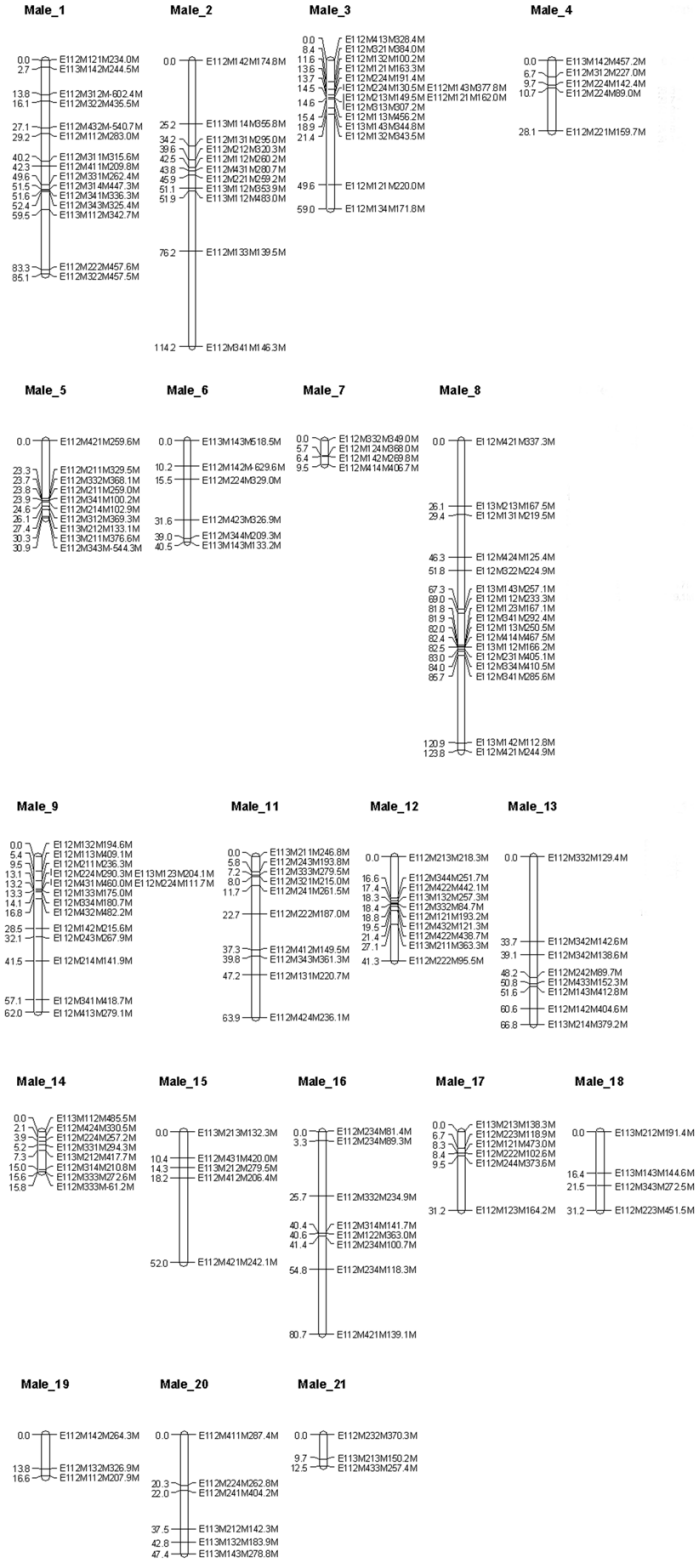
*Artemia franciscana* autosomal male linkage groups. Twenty linkage groups representing the *Artemia franciscana* autosomal genome containing markers originating from male parental strain San Francisco Bay (ARC1364). Each AFLP marker is represented by (1) a code referring to the corresponding PC (Table S1), followed by (2) the molecular size of the fragment in nucleotides and (3) the type of parental marker (male marker, tagged as “M”). Cumulative marker distances are indicated on the left (cM).

First, a parental map including only parental markers was constructed. Summary statistics for the parental maps are listed in Table 1. The grouping of parental markers at a LOD score ranging from 5.0 to 6.0 resulted in a number of linkage groups corresponding with the haploid chromosome number (n = 21). The female map, containing 225 markers (Figure 1), resulted in 22 “female” linkage groups (LG) spanning 1,312.9 cM; the male map, containing 181 markers (Figure 2), resulted in 21 “male” LG spanning 1,041.3 cM. Twenty-eight percent of the analyzed parental markers showed significant (*p*<0.05; χ^2^ test) segregation distortion. Male markers were more often distorted than female markers (31% resp. 25%). Some larger genomic regions did not contain any markers (e.g. 32.5 cM in LG Female_6, Figure 1; 38.0 cM in LG Male_2, Figure 2), despite the low median inter-marker distances of 3.9 and 3.1 cM for the female and the male linkage map (Table 1).

**Table 1:** Statistics for the female and male linkage maps.

		Female (Vinh Chau)	Male (San Francisco Bay)
No. of linkage groups		22	21
No. of markers mapped per linkage group	Min	3	3
	Max	19	17
	Median	12	8
	Mean	10	9
	Total	225	181
Size of linkage groups (cM)	Min	15.1	9.5
	Max	104.4	123.8
	Median	63.7	44.4
	Mean	59.7	52.1
	Total	1312.9	1041.3
Intermarker distance (cM)	Min	0.0	0.0
	Max	32.5	38.0
	Median	3.9	3.1
	Mean	6.5	6.6

**Figure 3:**
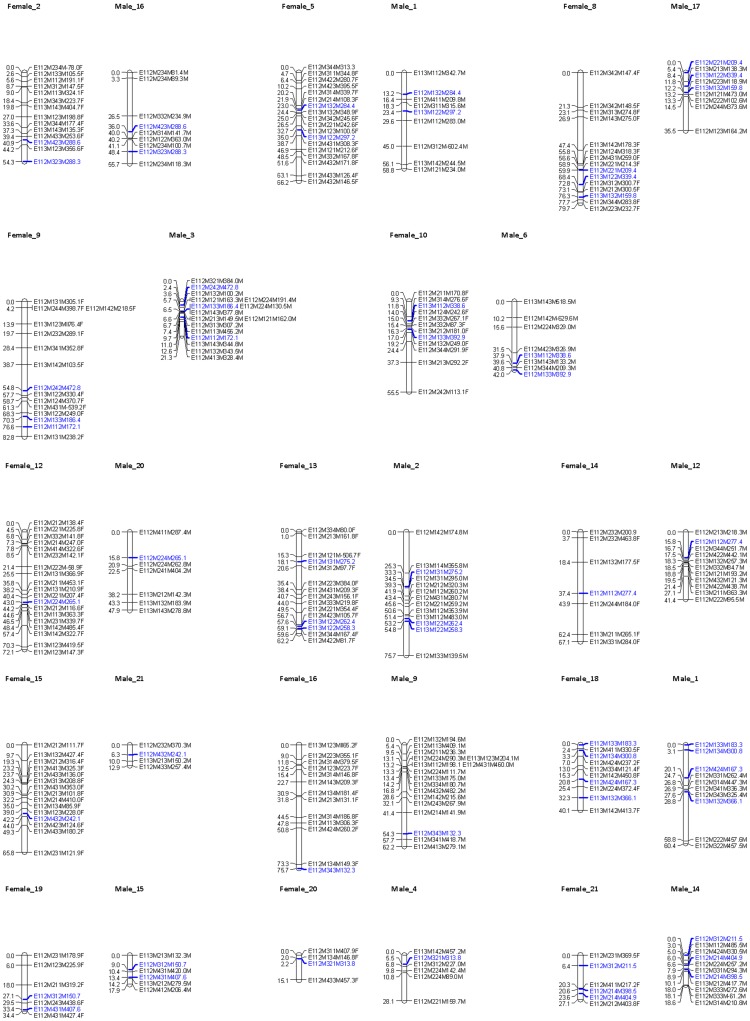
*Artemia franciscana* homologous autosomal male and female linkage groups. Fourteen homologous autosomal linkage group pairs. Each AFLP marker was identified by (1) a code referring to the corresponding PC (Table S1), followed by (2) the molecular size of the fragment in nucleotides and (3) the type of marker (female marker, tagged as “F”, male marker, tagged as “M”, biparental marker, no tag). Common biparental markers are indicated in blue. Cumulative marker distances are indicated on the left (cM).

**Figure 4:**
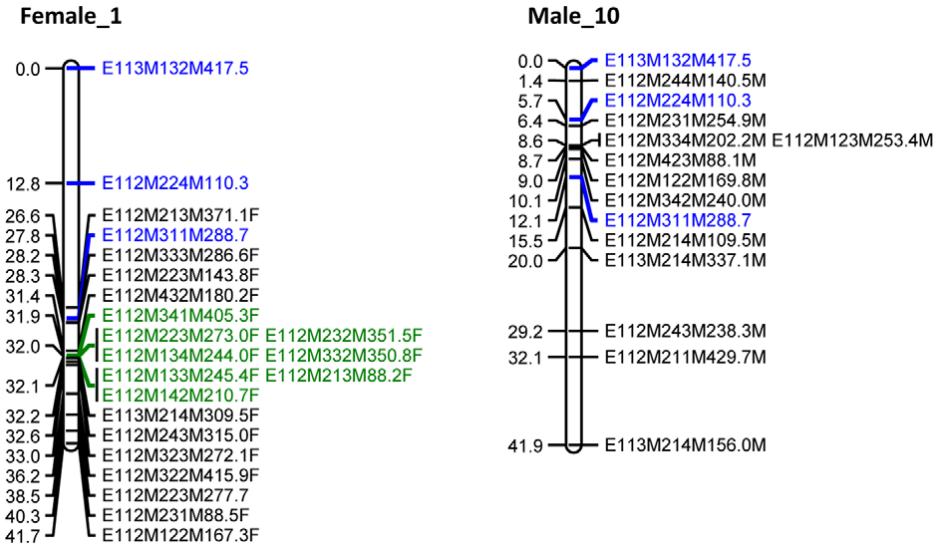
*Artemia franciscana* sex linkage groups. Female linkage group Female_1 corresponds with the W chromosome. The homologous male linkage group Male_10 corresponds with the Z chromosome. Each AFLP marker is represented by (1) a code referring to the corresponding PC (Table S1), followed by (2) the molecular size of the fragment in nucleotides and (3) the type of marker (female marker, tagged as “F”, male marker, tagged as “M”, biparental marker, no tag). Common biparental markers are indicated in blue. Markers fully linked to sex are marked in green. Cumulative marker distances (cM) are indicated on the left.

Next, an integrated map was created including the 98 biparental markers and 406 previously mapped parental markers (Figure 3). By including biparental markers, groups consistent with linkage groups of the parental map were obtained at a LOD threshold ranging between 6 and 10. Sixty-nine percent of the biparental marker loci showed significant (*p*<0.05; χ^2^ test) segregation distortion. These loci were still included in map construction and evaluated for quality afterwards, since significant segregation distortion is inherent to relatively small experimental mapping population sizes of ∼100 individuals. Forty-nine biparental markers (50%) could be mapped in the female as well as in the male map, identifying 15 homologous linkage groups including the sex linkage groups (Figure 3, Figure 4).

### Mapping of the sex locus

Staelens et al. [27] described segregation patterns of sex-linked AFLP markers that unequivocally differentiate the WZ-ZZ and XX-XY sex-determination system. We observed eight AFLP markers, spanning a region of 0.2 cM on LG Female_1 (markers in green, Figure 4) segregating according to pattern 1 and a single marker (E112M122M167.3F) according to pattern 2. Both segregation patterns are expected under the assumption of female heterogamety. None of the markers segregated according to patterns 6, 7 and 8, expected under the assumption of male heterogamety. The male linkage group Male_10 was identified as homologous to Female_1 (Figure 4). In conclusion, the observed segregation patterns of sex-linked AFLP markers strongly favour female over male heterogamety in *Artemia*.

### *A. franciscana* genome size estimation by flow cytometry

**Figure 5:**
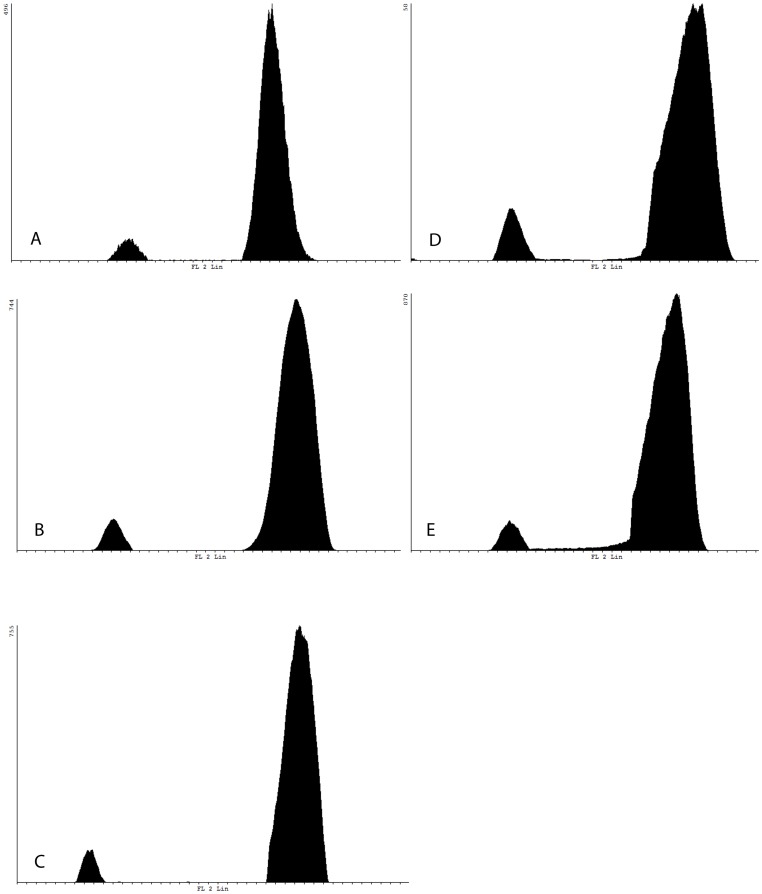
Fluorescence histograms for *Artemia franciscana* and chicken DNA content estimation. Fluorescence histogram of four different *A*. *franciscana* male individuals with trout as the internal standard (A, B, C, D) and of chicken CEN with trout as the internal standard (E).

Using trout blood as the internal standard, the haploid female chicken genome size (GS) determined by flow cytometry was 1.05 Gb (1.07 pg) as previously reported for female chicken [59]. We preferred rainbow trout nuclei as the internal standard in the assessment of the *Artemia* GS because their fluorescence values did not overlap with those of *Artemia*, as was the case with fluorescence values obtained from chicken nuclei. Using rainbow trout nuclei as the internal standard, the *A. franciscana* haploid genome size was estimated to 0.93±0.09 Gb (0.97±0.09 pg; n = 4). Fluorescence histograms for each sample and for chicken are shown in Figure 5. Fluorescence peaks were relatively broad due to cell debris from the previously frozen *Artemia* individuals, but average DNA content estimates were consistent throughout the different samples, shown by the small standard error.

## Discussion

We present the first sex-specific AFLP linkage maps and sex-linked markers as well as a consistent genome size (GS) estimation for the brine shrimp *A. franciscana*.

The linkage analysis of 433 parental AFLP markers segregating in a 112 full-sib family identified 21 male and 22 female linkage groups, corresponding very well with the haploid chromosome number in *A. franciscana* (2n = 42) [8]. Most likely, the markers in small linkage groups (LG) such as Female_20 (Figure 1) would join one of the other 21 LG by adding more markers to the female map. More female than male markers were generated, suggesting that maternal *A. franciscana* strain Vinh Chau (VC) has more unique alleles compared to the paternal strain San Francisco Bay (SFB). This seems a logical consequence of the SFB origins of VC. The level of polymorphism between the two *A. franciscana* parental strains was estimated at 36%, which is in the range of 9–50% estimated previously by Kappas et al. [53]. Given their high marker density, the produced genetic maps are adequate for the anchoring of *Artemia* genome sequences to facilitate the future construction of physical maps for each of the 21 chromosomes. This will be especially useful, considering the numerous reports of repetitive sequences in *Artemia* [63]. *Artemia* linkage maps will also allow future linkage studies in *Artemia* for important crustacean traits such as resistance to *Vibrio*, the most common bacterial pathogen in worldwide marine fish and shellfish aquaculture.

Fifteen homologous linkage groups, including the LG representing the sex chromosomes, were identified between the female and male linkage maps by including biparental markers in the linkage analysis. This study identified eight sex-linked AFLP marker alleles mapping to one locus and inherited from the female parent, suggesting *A. franciscana* adopts a genetic WZ-ZZ sex-determining system. *Artemia* sex-linked markers will enable the study of nauplii sex ratios and their dynamic in natural *Artemia* populations. They will also enable the further fine-mapping of the sex-determining locus and the subsequent identification of the primary sex-determining gene(s). Furthermore, based on sequence homology with *Artemia*, sex-determining genes might be identified in commercially valuable crustaceans, enabling PCR-based allele-specific assay development in the framework of the development of mono-sex cultures in shrimp [64].

The clustering of eight sex-linked markers in a 0.2 cM region suggests reduced recombination, which is often found in sex-linked regions [65]. Genes from a region that stopped recombining in the early evolution of sex chromosomes have a high sequence divergence, allowing an estimate of when the W and Z chromosomes first stopped recombining and thus, the age of the sex chromosome system [65].

The estimated *Artemia* GS in this study (0.93 Gb) is smaller than earlier estimates: 2.93 Gb by Feulgen densitometry [9] and 1.47 Gb by DNA reassociation kinetics [10]. “*A. salina”* used to be a general name for all *Artemia* species, presently confounding the identity of the investigated *Artemia* in many studies [1]. Because the *Artemia* DNA content measured by Feulgen densitometry on “*A. salina*” is almost a twofold of that measured by DNA reassociation kinetics, Feulgen densitometry might have been performed on a tetraploid *A. parthenogenetica*, as suggested by Vaughn [10]. Also, the absolute *A. franciscana* karyotype size varies between 60.68 µm and 139.26 µm [8], showing that significant intra-specific variation in DNA content could explain the high Feulgen densitometry values as well.

Vaughn [10] calculated the *Artemia* haploid GS by DNA reassociation kinetics, based on an *A. franciscana* GC content of 42%. More recent measurements however, show an *A. franciscana* (SFB) GC content of 32% determined by CsCl centrifugation and confirmed by direct chemical analysis and renewed thermal denaturation [61]. An estimated GC content lowered by 1% results in a 0.018% lower haploid DNA content estimated by DNA reassociation kinetics [66]. Hence, based on a GC content of 32%, the corrected *A. franciscana* DNA content estimated by Vaughn [10] is 1.23 Gb, approximating more closely the 0.93 Gb *Artemia* GS estimated in this study.

Currently, out of the 50,000 known Crustacea species, the GS of 278 crustaceans has been determined, covering a 400-fold-wide genome size range between *Cyclops kolensis*, a Cyclopoid copepod (0.14 pg) and *Ampelisca macrocephala*, an Arctic Amphipod (64.62 pg) [67], [68]. In comparison, *A. franciscana* has a relatively small genome of 0.97 pg. This makes it a potential new model crustacean for which genome sequencing is currently feasible, unlike for crustaceans with a much larger genome size. To date, the only publicly accessible sequenced crustacean genome is the branchiopod *D. pulex*, with an average genome size of 0.23 pg [69].

Ultimately, the further development of genomic resources for *Artemia* such as the whole genome sequence, will add a completely new dimension to *Artemia* research and its use as live food in aquaculture. Moreover, knowledge of the *A. franciscana* sex-determining system will facilitate future evolutionary studies of sex chromosomes in sexually dimorphic (WZ female/ZZ male) and parthenogenetic *Artemia.* Considering the presence of sexual and asexual reproduction strategies, the *Artemia* genus shows promise as a model system for the study of asexuality, its evolution and its evolutionary purpose. Finally, since *Artemia* is considered a potential crustacean model species, increasing knowledge about *Artemia* genetics and genomics in general and sex-related genetics in particular, are expected to be valuable to crustacean aquaculture, presently lacking in molecular breeding strategies despite their contribution of 23% to the total aquaculture production value [70].

## Supporting Information

Table S1 **List of the 65 primer combinations used for AFLP analysis^1^ E:** ***Eco*** **RI primer with three selective bases; M:** ***Mse*** **I primer with three selective bases (1, 2, 3, 4 correspond to A, C, G, T).**
(DOC)

## References

[1] Lavens P, Sorgeloos P (1996) Manual on the production and use of live food for aquaculture. FAO Fisheries Technical Paper 361; FAO, editor. Rome. 295 p.

[2] Kayim M, Ates M, Elekon HA (2010) The effects of different feeds under the same salinity conditions on the growth and survival rate of Artemia. Journal of Animal and Veterinary Advances 9: 1223–1226.

[3] Leger P, Bengtson DA, Simpson KL, Sorgeloos P (1986) The Use and Nutritional-Value of Artemia as a Food Source. Oceanography and Marine Biology 24: 521–623.

[4] Asem A, Rastegar-Pouyani N, De Los Ríos-Escalante P (2010) The genus *Artemia* Leach, 1819 (Crustacea: Branchiopoda). I. True and false taxonomical descriptions. Latin American Journal of Aquatic Research 38: 501–506.

[5] Browne RAB ST (1991) Taxonomy and population genetics of Artemia. Artemia biology. Boca Raton, FL.: CRC Press.

[6] Robbins HM, Van Stappen G, Sorgeloos P, Sung YY, MacRae TH, et al (2010) Diapause termination and development of encysted *Artemia* embryos: roles for nitric oxide and hydrogen peroxide. Journal of Experimental Biology 213: 1464–1470. 20400630 10.1242/jeb.041772

[7] Maniatsi S, Baxevanis AD, Kappas I, Deligiannidis P, Triantafyllidis A, et al (2011) Is polyploidy a persevering accident or an adaptive evolutionary pattern? The case of the brine shrimp *Artemia* . Molecular Phylogenetics and Evolution 58: 353–364. 21145977 10.1016/j.ympev.2010.11.029

[8] Parraguez M, Gajardo G, Beardmore JA (2009) The New World *Artemia* species *A. franciscana* and *A. persimilis* are highly differentiated for chromosome size and heterochromatin content. Hereditas 146: 93–103. 19490170 10.1111/j.1601-5223.2009.02109.x

[9] Rheinsmith EL, Hinegard R, Bachmann K (1974) Nuclear DNA amounts in crustacea. Comparative Biochemistry and Physiology, Part B 48: 343–348. 10.1016/0305-0491(74)90269-7 4847621

[10] Vaughn JC (1977) DNA reassociation kinetic analysis of brine shrimp, Artemia salina. Biochemical and Biophysical Research Communications 79: 525–531. 588285 10.1016/0006-291x(77)90189-9

[11] Libertini A, Trisolini R, Rampin M (2008) Chromosome number, karyotype morphology, heterochromatin distribution and nuclear DNA content of some talitroidean amphipods (Crustacea: Gammaridea). European Journal of Entomology 105: 53–58.

[12] Rees DJ, Belzile C, Glemet H, Dufresne F (2008) Large genomes among caridean shrimp. Genome 51: 159–163. 18356950 10.1139/g07-108

[13] Rees DJ, Dufresne F, Glémet H, Belzile C (2007) Amphipod genome sizes: first estimates for Arctic species reveal genomic giants. Genome 50: 151–158. 17546080 10.1139/g06-155

[14] Badaracco G, Bellorini M, Landsberger N (1995) Phylogenetic Study of Bisexual Artemia Using Random Amplified Polymorphic DNA. Journal of Molecular Evolution 41: 150–154. 7666444 10.1007/BF00170666

[15] Camargo WN, Bossier P, Sorgeloos P, Sun Y (2002) Preliminary genetic data on some Caribbean Artemia franciscana strains based on RAPD's. Hydrobiologia 468: 245–249.

[16] Bossier P, Xiaomei W, Catania F, Dooms S, Van Stappen G, et al (2004) An RFLP database for authentication of commercial cyst samples of the brine shrimp *Artemia* spp. (International Study on *Artemia* LXX). Aquaculture 231: 93–112.

[17] Sun Y SW-Q, Zhong Y-C, Zhang R-S, Abatzopoulos TJ, Chen R-Y (1999) Diversity and genetic differentiation in Artemia species and populations detected by AFLP markers. International Journal of Salt Lake Research 8: 10.

[18] Triantaphyllidis GV, Criel GRJ, Abatzopoulos TJ, Thomas KM, Peleman J, et al (1997) International Study on Artemia.57. Morphological and molecular characters suggest conspecificity of all bisexual European and North African Artemia populations. Marine Biology 129: 477–487.

[19] Munoz J, Green AJ, Figuerola J, Amat F, Rico C (2009) Characterization of polymorphic microsatellite markers in the brine shrimp Artemia (Branchiopoda, Anostraca). Molecular Ecology Resources 9: 547–550. 21564689 10.1111/j.1755-0998.2008.02360.x

[20] Stillman JH, Colbourne JK, Lee CE, Patel NH, Phillips MR, et al (2008) Recent advances in crustacean genomics. Integrative and Comparative Biology 48: 852–868. 21669837 10.1093/icb/icn096

[21] Valverde JR, Batuecas B, Moratilla C, Marco R, Garesse R (1994) The complete mitochondrial DNA sequence of the crustacean *Artemia franciscana* . Journal of Molecular Evolution 39: 400–408. 7966370 10.1007/BF00160272

[22] Weeks SC, Benvenuto C, Sanderson TF, Duff RJ (2010) Sex chromosome evolution in the clam shrimp, *Eulimnadia texana* . Journal of Evolutionary Biology 23: 1100–1106. 20298443 10.1111/j.1420-9101.2010.01963.x

[23] Ford AT (2008) Can you feminise a crustacean? Aquatic Toxicology 88: 316–321. 18550186 10.1016/j.aquatox.2008.04.013

[24] Parnes S, Khalaila I, Hulata G, Sagi A (2003) Sex determination in crayfish: are intersex *Cherax quadricarinatus* (Decapoda, Parastacidae) genetically females? Genetical Research 82: 107–116. 14768895 10.1017/s0016672303006372

[25] Juchault P, Rigaud T (1995) Evidence for female heterogamety in two terrestrial crustaceans and the problem of sex chromosome evolution in isopods. Heredity 75: 466–471.

[26] Alcivar-Warren A (2012) The plasticity of the shrimp genome -*sex*, retrotransposons, ribosomal RNAs, growth performance and disease susceptibility. Aquaculture America, International Marine Shrimp Environmental Genomics Initiative (IMSEGI) – Monitoring ecosystem, animal and public health. Las Vegas, Nevada.

[27] Staelens J, Rombaut D, Vercauteren I, Argue B, Benzie J, et al (2008) High-density linkage maps and sex-linked markers for the black tiger shrimp (*Penaeus monodon*). Genetics 179: 917–925. 18558652 10.1534/genetics.107.080150 PMC2429885

[28] Ventura T, Sagi A (2012) The insulin-like androgenic gland hormone in crustaceans: From a single gene silencing to a wide array of sexual manipulation-based biotechnologies. Biotechnology Advances 30: 1543–1550. 22561950 10.1016/j.biotechadv.2012.04.008

[29] Li YT, Dierens L, Byrne K, Miggiano E, Lehnert S, et al (2006) QTL detection of production traits for the Kuruma prawn Penaeus japonicus (Bate) using AFLP markers. Aquaculture 258: 198–210.

[30] Tomaszkiewicz M, Smolarz K, Wolowicz M (2010) Heterogamety in the Baltic Glacial Relict Saduria Entomon (Isopoda: Valvifera). Journal of Crustacean Biology 30: 757–761.

[31] Stefani R (1963) La digametia femminile in *Artemia salina* Leach e la constituzione del corredo cromosomico nei biotitic diploidi anfigonico e diploide partenogenético. Caryologia 16: 625–636.

[32] Bowen ST (1965) The genetics of *Artemia salina*. V. Crossing over between X and Y chromosomes. Genetics 52: 695–710. 5863914 10.1093/genetics/52.3.695 PMC1210933

[33] Cristescu MEA, Colbourne JK, Radivojac J, Lynch M (2006) A micro satellite-based genetic linkage map of the waterflea, *Daphnia pulex*: On the prospect of crustacean genomics. Genomics 88: 415–430. 16624519 10.1016/j.ygeno.2006.03.007

[34] Routtu J, Jansen B, Colson I, De Meester L, Ebert D (2010) The first-generation Daphnia magna linkage map. Bmc Genomics 11. 10.1186/1471-2164-11-508 PMC2997004 20860799

[35] Foley BR, Rose CG, Rundle DE, Leong W, Moy GW, et al.. (2011) A gene-based SNP resource and linkage map for the copepod Tigriopus californicus. Bmc Genomics 12. 10.1186/1471-2164-12-568 PMC3298550 22103327

[36] Maneeruttanarungroj C, Pongsomboon S, Wuthisuthimethavee S, Klinbunga S, Wilson KJ, et al (2006) Development of polymorphic expressed sequence tag-derived microsatellites for the extension of the genetic linkage map of the black tiger shrimp (Penaeus monodon). Animal Genetics 37: 363–368. 16879347 10.1111/j.1365-2052.2006.01493.x

[37] Wilson K, Li YT, Whan V, Lehnert S, Byrne K, et al (2002) Genetic mapping of the black tiger shrimp Penaeus monodon with amplified fragment length polymorphism. Aquaculture 204: 297–309.

[38] You EM, Liu KF, Huang SW, Chen M, Groumellec ML, et al (2010) Construction of integrated genetic linkage maps of the tiger shrimp (Penaeus monodon) using microsatellite and AFLP markers. Animal Genetics 41: 365–376. 20064148 10.1111/j.1365-2052.2009.02014.x

[39] Du ZQ, Ciobanu DC, Onteru SK, Gorbach D, Mileham AJ, et al (2010) A gene-based SNP linkage map for pacific white shrimp, Litopenaeus vannamei. Animal Genetics 41: 286–294. 19968647 10.1111/j.1365-2052.2009.02002.x

[40] Perez F, Erazo C, Zhinaula M, Volckaert F, Calderon J (2004) A sex-specific linkage map of the white shrimp Penaeus (Litopenaeus) vannamei based on AFLP markers. Aquaculture 242: 105–118.

[41] Zhang LS, Yang CJ, Zhang Y, Li L, Zhang XM, et al (2007) A genetic linkage map of Pacific white shrimp (Litopenaeus vannamei): sex-linked microsatellite markers and high recombination rates. Genetica 131: 37–49. 17043742 10.1007/s10709-006-9111-8

[42] Li ZX, Li J, Wang QY, He YY, Liu P (2006) AFLP-based genetic linkage map of marine shrimp Penaeus (Fenneropenaeus) chinensis. Aquaculture 261: 463–472.

[43] Wang W, Tian Y, Kong J, Li X, Liu X, et al (2012) Integration genetic linkage map construction and several potential QTLs mapping of Chinese shrimp (Fenneropenaeus chinensis) based on three types of molecular markers. Russian Journal of Genetics 48: 422–434. 22730771

[44] Li Y, Byrne K, Miggiano E, Whan V, Moore S, et al (2003) Genetic mapping of the kuruma prawn *Penaeus japonicus* using AFLP markers. Aquaculture 219: 143–156.

[45] Vainola R (1998) A sex-linked locus (Mpi) in the opossum shrimp Mysis relicta: implications for early postglacial colonization history. Heredity 81: 621–629.

[46] Mantovani B, Cesari M, Luchetti A, Scanabissi F (2008) Mitochondrial and nuclear DNA variability in the living fossil Triops cancriformis (Bosc, 1801) (Crustacea, Branchiopoda, Notostraca). Heredity 100: 496–505. 18285812 10.1038/hdy.2008.3

[47] Shuster SM, Levy L (1999) Sex-linked inheritance of a cuticular pigmentation marker in the marine isopod, Paracerceis sculpta Holmes (Crustacea: Isopoda: Sphaeromatidae). Journal of Heredity 90: 304–307.

[48] Siegismund HR (2002) Disparity in population differentiation of sex-linked and autosomal variation in sibling species of the Jaera albifrons (Isopoda) complex. Journal of Heredity 93: 432–439. 12642644 10.1093/jhered/93.6.432

[49] Gomez-Uchida D, Weetman D, Hauser L, Galleguillos R, Retamal M (2003) Allozyme and AFLP analyses of genetic population structure in the hairy edible crab Cancer setosus from the Chilean coast. Journal of Crustacean Biology 23: 486–494.

[50] Ventura T, Aflalo ED, Weil S, Kashkush K, Sagi A (2011) Isolation and characterization of a female-specific DNA marker in the giant freshwater prawn Macrobrachium rosenbergii. Heredity 107: 456–461. 21522169 10.1038/hdy.2011.32 PMC3199927

[51] Pannell JR (2008) Consequences of inbreeding depression due to sex-linked loci for the maintenance of males and outcrossing in branchiopod crustaceans. Genetics Research 90: 73–84. 18289402 10.1017/S0016672307008981

[52] Abatzopoulos TJ, Beardmore JA, Clegg JS, Sorgeloos P (2002) Artemia: basic and applied biology. Dordrecht: Kluwer Academic Publishers. 304 p.

[53] Kappas I, Abatzopoulos TJ, Van Hoa N, Sorgeloos P, Beardmore JA (2004) Genetic and reproductive differentiation of *Artemia franciscana* in a new environment. Marine Biology 146: 103–117.

[54] Hodgson R (1999) CTAB method for the isolation of total nucleic acid (TNA) from shrimp tissue. Workshop on “Molecular diagnostics for shrimp viruses in the Asian region”. Salaya.

[55] Vuylsteke M, Peleman JD, van Eijk MJT (2007) AFLP technology for DNA fingerprinting. Nature Protocols 2: 1387–1398. 17545976 10.1038/nprot.2007.175

[56] van Ooijen JW (2006) JoinMap® 4, Software for the calculation of genetic linkage maps in experimental populations. Wageningen: Kyazma B.V. 63 p.

[57] Voorrips RE (2002) MapChart: software for the graphical presentation of linkage maps and QTLs. Journal of Heredity 93: 77–78. 12011185 10.1093/jhered/93.1.77

[58] Genet C, Dehais P, Palti Y, Gao G, Gavory F, et al (2011) Analysis of BAC-end sequences in rainbow trout: Content characterization and assessment of synteny between trout and other fish genomes. BMC Genomics 12: 314. 21672188 10.1186/1471-2164-12-314 PMC3125269

[59] Mendonça MAC, Carvalho CR, Clarindo WR (2010) DNA content differences between male and female chicken (*Gallus gallus domesticus*) nuclei and Z and W chromosomes resolved by image cytometry. Journal of Histochemistry and Cytochemistry 58: 229–235. 19875846 10.1369/jhc.2009.954727 PMC2825488

[60] Doležel J, Bartoš J (2005) Plant DNA flow cytometry and estimation of nuclear genome size. Annals of Botany 95: 99–110. 15596459 10.1093/aob/mci005 PMC4246710

[61] Cruces J, Wonenburger MLG, Díaz-Guerra M, Sebastián J, Renart J (1986) Satellite DNA in the crustacean Artemia. Gene 44: 341–345. 3023196 10.1016/0378-1119(86)90200-3

[62] Zhou Y, Bizzaro JW, Marx KA (2004) Homopolymer tract length dependent enrichments in functional regions of 27 eukaryotes and their novel dependence on the organism DNA (G+C)% composition. Bmc Genomics 5. 10.1186/1471-2164-5-95 PMC539357 15598342

[63] Carettoni D, Landsberger N, Zagni E, Benfante R, Badaracco G (1994) Topoisomerase-I Action on the Heterochromatic DNA from the Brine Shrimp Artemia-Franciscana – Studies in-Vivo and in-Vitro. Biochemical Journal 299: 623–629. 8192650 10.1042/bj2990623 PMC1138066

[64] Ventura TA, E.D Sagi (2009) A (2009) Future prospects of crustacean monosex culture: could giant prawn monosex culture benefit from the discovery of an insulin-like factor? Aquaculture Europe 34: 30–31.

[65] Bergero R, Charlesworth D (2009) The evolution of restricted recombination in sex chromosomes. Trends in Ecology & Evolution 24: 94–102. 19100654 10.1016/j.tree.2008.09.010

[66] Seidler RJ, Mandel M (1971) Quantitative aspects of deoxyribonucleic acid renaturation: base composition, state of chromosome replication, and polynucleotide homologies. Journal of Bacteriology 106: 608–614. 4929869 10.1128/jb.106.2.608-614.1971 PMC285137

[67] Gregory TR (2005) Animal Genome Size Database.

[68] Jeffery NW (2012) The first genome size estimates for six species of krill (Malacostraca, Euphausiidae): large genomes at the north and south poles. Polar Biology 35: 959–962.

[69] Vergilino R, Belzile C, Dufresne F (2009) Genome size evolution and polyploidy in the Daphnia pulex complex (Cladocera: Daphniidae). Biological Journal of the Linnean Society 97: 68–79.

[70] Benzie JAH (2009) Use and exchange of genetic resources of penaeid shrimps for food and aquaculture. Reviews in Aquaculture 1: 232–250.

