# Supplementary material for: A first AFLP-Based Genetic Linkage Map for Brine Shrimp Artemia franciscana and Its Application in Mapping the Sex Locus
Source: PLoS One. 2013 Mar 4;8(3):e57585. doi: 10.1371/journal.pone.0057585 (PMC3587612; doi:10.1371/journal.pone.0057585)
Supplement: Table S1 — List of the 65 primer combinations used for AFLP analysis1 E: Eco RI primer with three selective bases; M: Mse I primer with three selective bases (1, 2, 3, 4 correspond to A, C, G, T). (DOC) [file pone.0057585.s001.doc]

| E112M112 | E112M222 | E112M323 | E112M431 |
| --- | --- | --- | --- |
| E112M113 | E112M223 | E112M331 | E112M432 |
| E112M121 | E112M224 | E112M332 | E112M433 |
| E112M122 | E112M231 | E112M333 | E113M112 |
| E112M123 | E112M232 | E112M334 | E113M114 |
| E112M124 | E112M233 | E112M341 | E113M122 |
| E112M131 | E112M234 | E112M342 | E113M123 |
| E112M132 | E112M241 | E112M343 | E113M132 |
| E112M133 | E112M242 | E112M344 | E113M142 |
| E112M134 | E112M243 | E112M411 | E113M143 |
| E112M142 | E112M244 | E112M412 | E113M211 |
| E112M143 | E112M311 | E112M413 | E113M212 |
| E112M211 | E112M312 | E112M414 | E113M213 |
| E112M212 | E112M313 | E112M421 | E113M214 |
| E112M213 | E112M314 | E112M422 |  |
| E112M214 | E112M321 | E112M423 |  |
| E112M221 | E112M322 | E112M424 |  |
